# AI-based body composition score predicts survival after liver transplantation

**DOI:** 10.1007/s00423-025-03885-4

**Published:** 2025-10-22

**Authors:** Eugen Malamutmann, Friederike Roehrborn, Ksenia Vershinina, Sven Koitka, Derar Jaradat, Sophia M. Schmitz, Johannes Haubold, Ulf P. Neumann, Felix Nensa, Arzu Oezcelik

**Affiliations:** 1 Department of General, Visceral and Transplantation Surgery, University Medicine Essen, Hufelandstraße 55, Essen, 45147 Germany; 2 Institute of Artificial Intelligence, University Medicine Essen, Essen, Germany; 3 Institute of Radiology and Neuroradiology, University Medicine Essen, Essen, Germany; 4 Department of Surgery, University Hospital Graz, Graz, Austria; 5 Institute of Sex and Gender Sensetive Medicine, University Medicine Essen, Essen, Germany

**Keywords:** Body composition, Liver transplantation, Sarcopenia, Frailty

## Abstract

**Purpose:**

Body composition has a significant role to predict survival in patients with malignant disease. This study evaluates the importance of body composition for predicting short- and long-term survival after liver transplantation. Additionally, the sex specific differences will be evaluated.

**Methods:**

Body composition, of all patients who underwent liver transplantation between January 2011 and December 2023 with computed tomography prior liver transplantation, was assessed fully automated with AI based technique. Pre-, intra- and postoperative data were retrospectively reported. Uni- and multivariate regression analyses was performed to identify independent prognostic factors for survival. The statistical analyses was performed separately for male and female with comparison of the both groups.

**Results:**

There were 346 patients (60.1%male, 39.9%female) with median age of 52.2 years (SD 10.8) included to the study. The univariate and multivariate cox regression analyses have identified the ratio of the subcutaneous fat volume to muscle volume as well as the ratio of the visceral fat volume to muscle volume as significant prognostic parameter for the overall survival. The separate analyses of the two groups show that these factors predict survival in male and female. However, visceral fat and also the ratio of FVM is significantly higher in male.

**Conclusion:**

Based on the results of our study we can conclude that the ratio of visceral fat volume to muscle volume (FVM-ratio) has an essential impact on overall survival after liver transplantation in male and female patients. The fully automated AI based assessment is fast, accurate and investigator independent.

## Introduction

 Organ shortage is a well-known problem in transplantation medicine worldwide, particularly in Germany. Consequently, optimal patient selection is becoming increasingly important to improve short- and long-term survival. Organ allocation was based on the MELD (Model of End Stage liver Disease) score, which reflects the urgency of liver transplantation (LT). However, the prognostic predictive value of outcomes after LT is limited. Besides many other well-known parameters, several recent studies have clearly shown a significant impact of psoas muscle volume as a parameter of sarcopenia as well as visceral adipose tissue volume on the outcome after LT [1]. While the influence of each factor is evident, the role of the relation of the psoas muscle to visceral adipose tissue, such as `Sarcopenic Obesity,’ for survival after LT is not adequately studied. It is likely that there were significant sex differences in body composition. However, there are no clear data for liver transplant patients. Preoperative identification of these parameters as prognostic factors, which increase the risk for postoperative complications or inferior outcome, would allow more tailored postoperative care. An accurate assessment of psoas and adipose tissue volumes is essential to address this question. Precise and objective calculations of these factors are time-consuming and extremely difficult. The need for new tools to easily implement these factors in the preoperative evaluation process of the patients for LT is widely accepted. In a previous study, we developed a fully automated body composition analysis method for routine CT imaging using convolutional neural networks in a previous study. We assessed the exact volume of total muscle and subcutaneous and intra-abdominal adipose tissue (SAT and VAT). Fully automated volume assessments are examiner-independent, reproducible, and fast [2]. This study aimed to evaluate body composition, which includes subcutaneous and visceral adipose tissue volume, muscle volume, and the ratio of adipose tissue to muscle volume, as a prognostic factor for the outcome after LT in adult patients. Additionally, the evaluation should identify sex-based differences between male and female patients regarding the effect of body composition on survival.

## Patients and methods

All patients who underwent deceased-donor LT between January 2011 and December 2023 were retrospectively reviewed. Patients who had computed tomography (CT) images in the electronic system of our hospital less than three months before LT were identified and included in the study. Pediatric patients, those with acute liver failure, and those who underwent re-transplantation were excluded. Preoperative, intraoperative, and postoperative data were retrospectively reported from our hospital’s electronic database and access to the patients’ medical records.

**Fig. 1:**
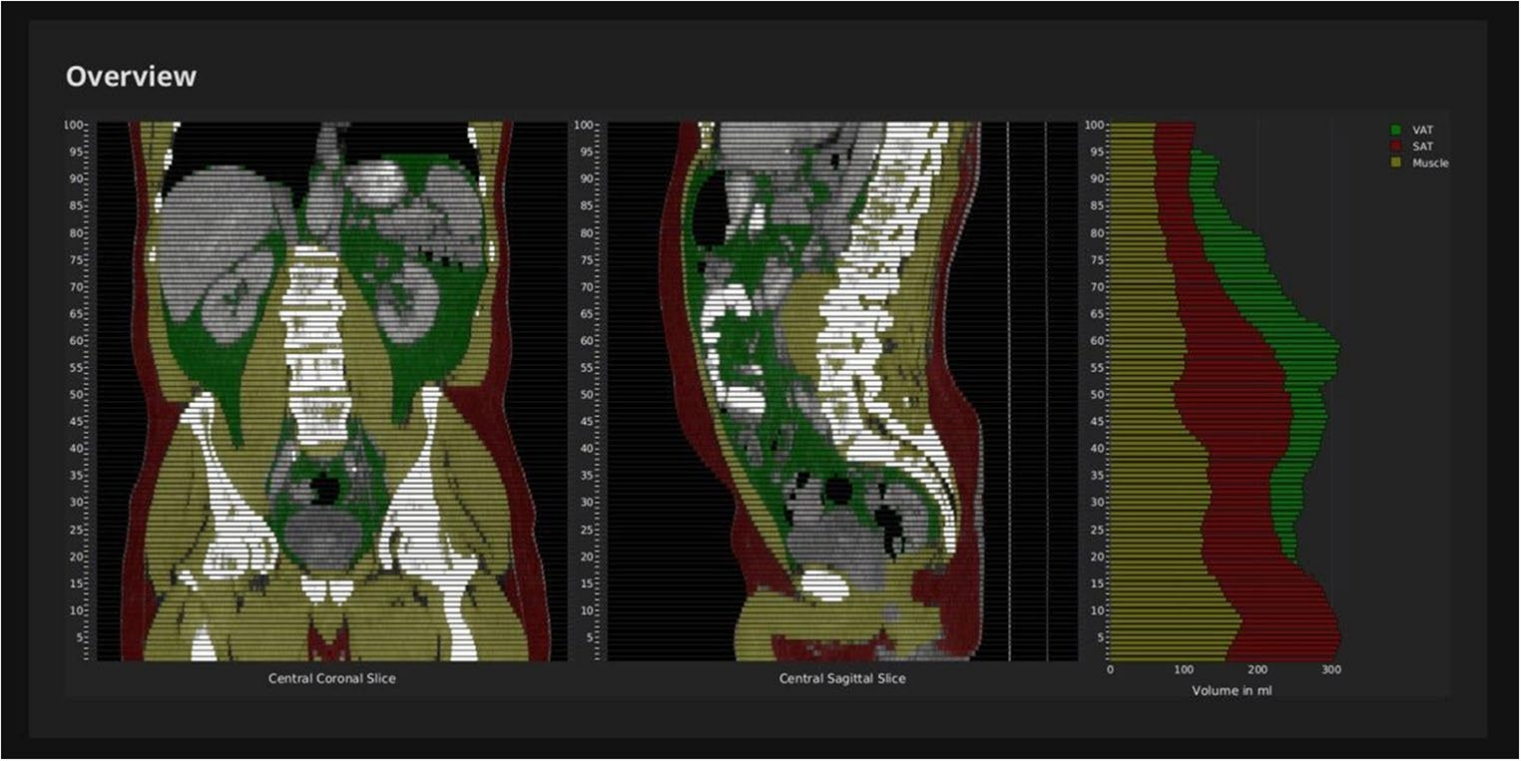
CT-Abdomen for the assessment of the total volume of subcutaneous- and visceral adipose tissue and muscle tissue using convolutional neuronal network

**Fig. 2:**
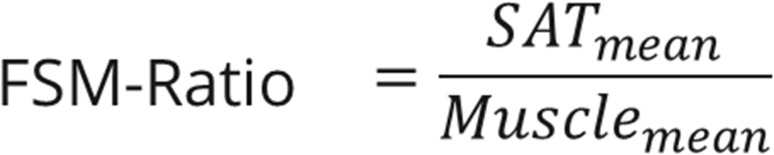
FSM-Ratio = Ratio of the mean subcutaneous fat volume to the mean muscle volume, SAT = subcutaneous adipose tissue

**Fig. 3:**
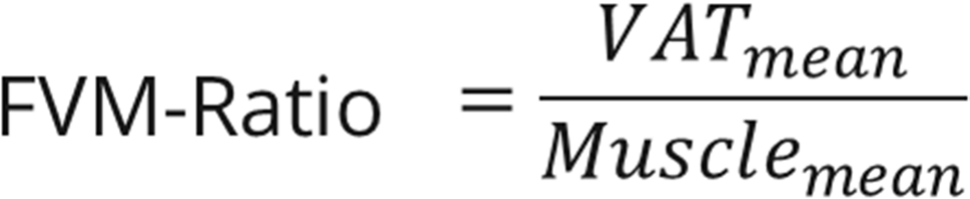
FVM-Ratio = Ratio of the mean visceral fat volume to the mean muscle volume, VAT = Visceral adipose tissue

Abdominal computed tomography (CT) was performed from the diaphragm to thigh. Triple-phase, contrast-enhanced, multidetector abdominal computed tomography (CT) with a slice thickness of 5 mm was performed. All CT scans were performed using multidetector-row systems, mainly with 16 or more detector-row systems. Venous phase imaging was performed 70–80 s after the intravenous administration of a contrast agent, with a median tube voltage of 115 kVp, ranging from 90 to 150 kVp. The total muscle, SAT, and VAT volumes on these CT examinations were assessed using a neuronal network, as described in our previous study (Fig. 1**)** [2]. In addition, the ratios of subcutaneous adipose tissue volume to muscle volume (FSM), visceral adipose tissue volume to muscle volume (FVM) (Figs. 2 and 3), and total adipose tissue volume to muscle volume were calculated.

All patients underwent orthotopic deceased-donor LT, using a standard technique with caval interposition. Intraoperative and postoperative data, such as the duration of surgery, blood transfusion, cold ischemic time, warm ischemic time, duration of intensive care unit (ICU) stay, postoperative complications, duration of hospital stay, and survival rates were reported. Perioperative mortality was defined as mortality within 30 days after liver transplantation. Immunosuppressant therapy was based on calcineurin inhibitors and prednisone supplemented with mycophenolate mofetil. Modifications in dose or compounds were performed individually depending on the clinical course. All patients were followed-up after LT according to the standard follow-up protocol. Morbidity was defined according to the Clavien– Dindo classification of surgical complications, with severe postoperative complications defined as grades IIIb and IV [3]. 

This study was conducted in accordance with the principles of the World Medical Association as defined in the Declaration of Helsinki and the national regulations for the conduct of clinical trials. Institutional review board approval was obtained from all participants.

### Statistical analyses

Values are reported as median values and interquartile ranges (IQR) or as mean values and standard deviations. We used the Mann– Whitney test to compare continuous variables and Fisher’s exact test or the chi-square test to compare proportions. Univariate, multivariate regression, and linear multivariate analyses were performed to identify independent prognostic factors for overall survival after LT. They were tested in different models because BMI, SAT, VAT, and muscle volume are integrative variables. Statistical analyses were performed for the whole group, separately for female and male patients, and these two groups were compared. Statistical significance was defined as p *<* 0.05. A professional biostatistician used the Statistical Package for the Social Sciences version 23.0.

## Results

Between January 2011 and December 2023, 1091 patients with end-stage liver disease underwent liver transplantation (LT) at our center. Of these, 346 patients identified with CT studies in our hospital’s electronic system less than three months before LT were included in the study. There were 208 male (60.1%) and 138 female (39.9%) patients, with a mean age of 52.2 years (SD 10.8). The mean BMI was 26.4 kg/m^2^ (SD 4.8). The main etiologies of cirrhosis were hepatitis C virus infection (105 patients, 30.3%) and alcoholic liver cirrhosis (99 patients, 28.6%). Comorbidities were observed in 287 (82.9%) patients, cardiovascular disease in 141 (40.4%), diabetes mellitus in 112 (32.4%), and renal disease in 98 (28.3%). The mean MELD score at the time of the LT was 24.3 (SD 5.0) **(**Table 1**)**. A comparison of male and female patients showed that male patients were significantly older than female patients (55 vs. 51 years, *p* < 0.03). As expected, BMI was significantly higher in male patients (26.4 vs. 22.7, *p* < 0.001). The distribution of etiology was significantly different between the two groups (*p* < 0.001). Diabetes and coronary artery disease as comorbidities were significantly more common in male patients (*p* < 0.01, *p* < 0.001). The mean volumes of the subcutaneous and visceral adipose tissue and muscle tissue were assessed as fully automated by AI, and the calculated ratio of subcutaneous adipose tissue to muscle volume and visceral adipose tissue to muscle volume are shown in Table 2. A comparison of male and female patients showed significant differences between the two groups. The volume of subcutaneous adipose tissue was 12% higher than that in male patients (*p* < 0.02). The volume of visceral adipose tissue in male was 54.2% higher than female patients (*p* < 0.0001). The total muscle volume of male was 30.8% higher than that of female. The ratio of FSM was 47.9% higher in female patients (*p* < 0.0001). The ratio of FVM is 22.0% higher in male than in female (*p* < 0.0001).

**Table 1:** Demographic data

*n* = 346	
Mean age (years (SD))	52.2 (10.8)
Sex	208 male (60.1%), 138 female (39.9%)
Mean body mass index (SD)	26.4 kg/m (SD 4.8)
Mean MELD score (SD)	24.3 (SD 5.0)
Co-morbidities (n (%))	287 (82.9%)

SD = standard deviation, MELD = Model of End Stage liver Disease

**Table 2:** Mean values of body composition

Variables	Mean	SD
Subcutaneous adipose tissue volume (SAT)	83.6 ml	47.9 ml
Visceral adipose tissue volume (VAT)	52.0 ml	29.9 ml
Muscle Volume	68.1 ml	14.7 ml
FSM-ratio	1.23	0.71
FVM-ratio	0.76	0.41

FSM = Ratio of subcutaneous fat to muscle volume; FVM = Ratio of visceral fat to muscle volume

The mean cold ischemic time was 448 min (SD: 139.5), and the mean warm ischemic time was 33 min (SD: 17.6). Blood transfusions were necessary in 177 patients (51.1%). The mean number of blood transfusions was 2.5 units (SD 1.7). The mean duration of surgery was 306 min (SD, 108). All the patients were observed postoperatively in the ICU. Major postoperative complications were observed in 86 (24.8%) patients. These were biliary complications with interventional or surgical treatment in 32 patients (9.2%), postoperative bleeding in 36 patients (10%), arterial complications with surgical treatment in nine patients (2.6%), and other complications in nine patients (2.6%). Perioperative mortality (30 days mortality) was observed in 46 (13.2%) patients. The median ICU stay was 11.0 days (SD, 6.0). The mean hospital stay was 30 days (SD 18). The median overall survival (OS) was 146 months. The 1-, 3-, and 5-year survival rates were 87%, 83%, and 74%, respectively.

The mean time to surgery and the mean warm ischemic time were significantly shorter in female patients than in male patients (235 vs. 269 min, *p* < 0.001 and 30 vs. 27 min, *p* < 0.001, respectively). No significant differences were observed in any of the other parameters, including survival.

The results of univariate and multivariate Cox regression analyses are presented in Table 3. The ratio of FSM and the ratio of FVM were identified as significant prognostic factors for OS. Parameters such as subcutaneous fat volume, visceral fat volume, muscle volume, and BMI were not significant predictors for OS. Of all the significant factors, the FVM-ratio had the most significant impact on OS. Separate univariate and multivariate Cox regression analyses of female and male patients identified the same factors as significant predictive factors for OS.

**Table 3:** Univariate and multivariate Cox regression analyses for survival

	Univariate analyses			Multivariate analyses		
Variables	HR	*p*-value	CI	HR	*p*-value	CI
BMI	1.02	0.09	1.00-1.05	1.02	0.09	1.00−1.05
MELD-Score	1.04	0.001	1.01–1.06	1.02	0.08	1.00−1.04
Cold ischemic time	1.0	0.355	1.00-1.01	1.00	0.45	1.00–1.00
Time of Surgery	1.0	< 0.001	1.00-1.02	1.00	< 0.001	1.00–1.00
Blood transfusion	1.10	0.001	1.05–1.15	1.07	< 0.001	1.03–1.10
Subcutaneous adipose tissue volume (SAT)	1.0	0.277	1.00-1.01	1.00	0.16	1.00–1.00
Visceral adipose tissue volume (VAT)	1.01	0.210	1.00-1.02	1.01	0.35	1.00−1.01
Muscle Volume	1.00	0.564	0.98–1.01	1.00	0.96	0.99–1.01
FSM-ratio	1.45	0.016	1.07–1.96	1.12	0.04	0.88–1.41
FVM-ratio	2.31	0.023	1.12–4.73	1.96	0.03	1.05–3.64

FSM = Ratio of subcutaneous fat to muscle volume; FVM = Ratio of visceral fat to muscle volume

## Discussion

Several retrospective studies have clearly shown that sarcopenia, as well as subcutaneous and visceral adipose tissue volume, have a significant impact on the mortality rate of patients on the waitlist for liver transplantation (LT) and on the posttransplant period [4–7]. According to recent studies, it is likely that the relationship between these compartments plays a more critical role than the absolute value of each compartment [8, 9]. Engelmann et al. assessed in their retrospective study the body composition of patients with liver cirrhosis listed for LT and those who underwent liver transplantation. A total of 612 patients who underwent abdominal CT at the time of transplantation were included in the study. Body composition, such as subcutaneous fat, visceral fat, and psoas muscle volume, was determined manually using the SliceOmatic software to quantify different tissue compartments in cross-sectional images based on Houndsfield unit thresholds. Cox regression analyses showed that decreased subcutaneous fat was associated with an increased risk of cirrhosis-related complications and death on the waitlist. Increasing paraspinal and visceral fat were positively correlated with metabolic comorbidities before transplantation and were predictive of 1-year survival after transplantation.

Consequently, the distribution of body fat is a significant determinant of the outcome [10]. Similar results were observed in our previous retrospective and prospective studies that evaluated the roles of sarcopenia and frailty [1].

The impact of body composition on post-transplant mortality after LT was addressed in another recently published study. They included 116 patients who underwent LT and measured subcutaneous and visceral fat and psoas muscle volume manually on abdominal CT scans. Patients who met the criteria for visceral obesity and sarcopenia were defined as those with sarcopenic visceral obesity. The results showed significantly lower survival rates in the patients with sarcopenic obesity. This difference in survival was not observed in patients who met only the criteria for sarcopenia or visceral obesity. The relative distribution of adipose tissue is more important than its absolute value [11]. The results of our study confirm these findings. Our study showed that the ratio of visceral adipose tissue volume to muscle volume (FVM) is the strongest prognostic factor for survival after liver transplantation. The FVM-ratio increases if the visceral fat volume is higher than the skeletal muscle volume, which leads to decreased survival. To the best of our knowledge such a score to define the relation of body compositions was not described in previous studies. We calculated the FVM- and FSM score for the first time and could show its significance for survival after LT.

Consequently, the relationship between visceral fat volume and skeletal muscle volume was more relevant than the absolute values for visceral fat and skeletal muscle volume. Even patients with a normal BMI have inferior survival rates if their weight is mainly based on visceral fat volume rather than on muscle volume. Accurately assessing visceral fat and muscle volume is essential for predicting survival after LT. Several methods have been described in the literature to assess body composition. The gold standard is the use of cross-sectional CT-derived assessment [1, 8, 12]. All these methods of body composition assessment have limitations. There is a lack of recommendations for technical standards such as contrast agents, tube voltage, and slide thickness. Another problem is the discriminating ability between muscle/soft tissue compartments in patients with end-stage liver disease, which leads to strong investigator dependency.

Another major problem is that all manual and semi-automated segmentation tools are time-consuming and labor-intensive [13]. The fully automated AI-based body composition assessment method used in our study is investigator-independent, requires technical standards, and can be performed in seconds, as we have proven previously [2]. However its impact on survival after LT was not evaluated before. In this study we used for the first time the fully automated AI based tool on large number of patients to determine body composition parameters. Our results have shown clearly that the AI based assessed body composition has significant impact on survival. This study showed that this method can also be used in patients with end-stage liver disease to predict survival after LT.

Recent studies have described myosteatosis as a significant factor for survival after major liver resection for malignant disease and for survival prediction after LT [14, 15]. Myosteatosis was assessed using HU thresholds on the CT scans. The discrimination of normal muscles and muscles with signs of myomatosis using this method is even more difficult than that using regular body composition. The fully automated AI-based assessment method can also describe and define standards for muscle quality, which can be correlated with muscle strength measured using handgrip strength.

The impact of sex/gender on body composition is obvious and addressed in many studies in different areas of medicine. The sex/gender specific impact of body composition of the outcome after liver transplantation has not been studied before. There are no data in the literature which address this question. Our study evaluates sex/gender specific differences, especially body composition factors and their impact on survival. As expected, there are significant differences between male and female patients regarding the volume of subcutaneous- and visceral fat tissue and also the muscle volume. Additionally we could show that the FVM ratio is significantly higher in female patients compared to male patient. In the multivariate analyses is the FVM ratio a independent prognostic factor in male and female. However, the difference in FVM ratio does not lead to significantly different survival data in our patients population.

The main limitation of our study was its retrospective nature and drawbacks. The large number of patients required to reach solid conclusions for a new method is difficult to achieve in a prospective design. Another limitation is selection bias due to the inclusion criteria. Only patients who underwent CT 30 days before transplantation were included in the study. We can assume that patients who require a CT scan are probably in reduced general condition and may have clinical complications.

Consequently, our study population comprised patients who underwent LT under reduced conditions. However, because we did not compare the groups, selection bias did not impact the significance of our results for this study population.

In addition, selecting the right patients, particularly those with reduced conditions, is of great importance.

## Conclusion

Based on the results of our study, we can conclude that the ratio of subcutaneous fat volume to muscle volume, as well as the ratio of visceral fat volume to muscle volume, is an independent prognostic factor for overall survival after LT. Although the body composition is significantly different between female and male patients, the prognostic factors are the same in both groups. Accurate manual assessment of volumes is challenging because it is time-consuming and investigator-dependent. A completely automated AI-based assessment is fast and investigator-independent.

## Data Availability

No datasets were generated or analysed during the current study.
