# Correction to: AI-based body composition score predicts survival after liver transplantation

**DOI:** 10.1007/s00423-025-03930-2

**Published:** 2025-11-21

**Authors:** Eugen Malamutmann, Friederike Roehrborn, Ksenia Vershinina, Sven Koitka, Derar Jaradat, Sophia M. Schmitz, Johannes Haubold, Ulf P. Neumann, Felix Nensa, Arzu Oezcelik

**Affiliations:** 1 Department of General, Visceral and Transplantation Surgery, University Medicine Essen, Hufelandstraße 55, 45147 Essen, Germany; 2 Institute of Artificial Intelligence, University Medicine Essen, Essen, Germany; 3 Institute of Radiology and Neuroradiology, University Medicine Essen, Essen, Germany; 4 Department of Surgery, University Hospital Graz, Graz, Austria; 5 Institute of Sex and Gender Sensetive Medicine, University Medicine Essen, Essen, Germany

**Correction to: Langenbeck's Archives of Surgery (2025) 410:307**

 10.1007/s00423-025-03885-4

 The original version of this article unfortunately contained mistakes. The author’s name, Derar Jaradat, was misspelled as Derar Jaradad.

 Furthermore, Derar Jaradat’s affiliations have been updated to reflect the following:

 Department of Surgery, University Hospital Graz, Graz, Austria

Department of General, Visceral and Transplantation Surgery, University Medicine Essen, Germany

The original article has been corrected.

